# Role of TNF-α in lung tight junction alteration in mouse model of acute lung inflammation

**DOI:** 10.1186/1465-9921-8-75

**Published:** 2007-10-30

**Authors:** Emanuela Mazzon, Salvatore Cuzzocrea

**Affiliations:** 1 IRCCS Centro Neurolesi "Bonino-Pulejo", Messina, Italy; 2 Department of Clinical and Experimental Medicine and Pharmacology, School of Medicine, University of Messina, Italy

## Abstract

In the present study, we used tumor necrosis factor-R1 knock out mice (TNF-αR1KO) to understand the roles of TNF-α on epithelial function in models of carrageenan-induced acute lung inflammation. In order to elucidate whether the observed anti-inflammatory status is related to the inhibition of TNF-α, we also investigated the effect of etanercept, a TNF-α soluble receptor construct, on lung TJ function. Pharmacological and genetic TNF-α inhibition significantly reduced the degree of (1) TNF-α production in pleural exudates and in the lung tissues, (2) the inflammatory cell infiltration in the pleural cavity as well as in the lung tissues (evaluated by MPO activity), (3) the alteration of ZO-1, Claudin-2, Claudin-4, Claudin-5 and β-catenin (immunohistochemistry) and (4) apoptosis (TUNEL staining, Bax, Bcl-2 expression). Taken together, our results demonstrate that inhibition of TNF-α reduces the tight junction permeability in the lung tissues associated with acute lung inflammation, suggesting a possible role of TNF-α on lung barrier dysfunction.

## Introduction

An important consequence of acute lung injury is the disruption of the paracellular alveolar permeability barrier [1]. The permeability barrier in terminal airspaces of the lung is due in large part to tight junctions between alveolar epithelial cells, which regulate the flow of molecules between apical and basolateral compartments [2,3]. Transmembrane proteins in the occludin and claudin families are the major transmembrane structural elements of tight junctions [4,5]. It has previously been shown that alveolar epithelial cells express occludin and zona occludens 1 (ZO-1) as part of the tight junction complex [6,7]. In addition to these components, the importance of claudins in pulmonary barrier function is underscored by the viability of occludin-deficient mice [8].

Moreover, is well known that airway epithelial cells perform many important functions, serving as an interface between environmental stimuli and the lung parenchyma. Normally the lower airways are pristine, free of bacterial flora or inflammatory cells, and are well protected by several layers of defenses including antimicrobial peptides, mucin, and ciliary action. There is a brisk epithelial response to airway injury caused by many different mechanisms [9,10]. Acute lung inflammatory response is also is associated to epithelial cytokine expression [11] as well as to the expression of the signaling cascade leading to apoptosis (programmed cell death). Activation of epithelial proinflammatory signaling cascades is mediated by tumor Necrosis Factor (TNF)-α a prototypic member of a cytokine family which regulates essential biologic functions (e.g. cell differentiation, proliferation, survival, apoptosis) and a broad spectrum of responses to stress and injury [12]. It is primarily produced by immune cells such as monocytes and macrophages, but it can also be released by many other cell types, including acinar cells. Membrane bound or soluble TNF-α interacts with two different surface receptors, TNF-α receptor 1 (TNFR1), or p55, and TNF-α receptor 2 (TNFR2), or p75 [13]. Although the extracellular domains of TNFR1 and TNFR2 are homologous and manifest similar affinity for TNF-α, the cytoplasmic regions of the two receptors are distinct and mediate different downstream events. Although most cell lines and primary tissues express both isoforms [14], most of the biological activities of TNF-α are mediated through TNF-R1 [15]. TNF-R2 is a poor inducer of apoptosis [16] and binding affinities of soluble TNF-a are significantly higher to TNF-R1 [15].

After exposure to TNF-α, target cells may down-regulate their responsiveness to the cytokine by shedding the receptors into the circulation. A natural mechanism which has been hypothesized to counteract excessive concentrations of circulating TNF-α (and the subsequent enhanced surface receptor activation) is the release of soluble receptors. The two soluble receptor forms (sTNFR1 and sTNFR2) have longer half lives than TNF-α, and their concentration may reflect TNF-α activity [17].

A primary role for TNF-α in inflammatory process (e.g. sepsis, endotoxiemic shock and acute pancreatitis) is suggested by several studies conducted upon cell lines, animal models and human beings [18-20]. In inflammation, over-production of TNF-α is pivotal in the induction of inflammatory genes, cell death, endothelial up-regulation and in the recruitment and activation of immune cells [21,22]. It has been also regarded as one of the major mediators of systemic progression and tissue damage in severe disease. However, the biologic significance of TNFR shedding is unclear. It could represent a neutralizing mechanism to counteract excessive TNF-α activity, but – on the other hand – it has been suggested that in relatively low concentrations sTNFR may serve as carriers to distant organs. Furthermore, sTNFR stabilize TNF-α trimeric structure thereby prolonging its half-life and augmenting its biological effects [17]. Etanercept is a fully humanized dimeric soluble form of the p75 TNF receptor that can bind to two TNF-α molecules blocking their interaction with cell surface TNFRs and rendering TNF-α biologically inactive. TNF-α inactivation is one thousand times stronger than inactivation by p75 monomeric TNFR [23,24]. It inhibits the activity of TNF-α *in vitro *and has been examined *in vivo *for its effects in different animal model systems of inflammatory and autoimmune diseases [25].

In addition, it has been demonstrated that TNF plays a role in the control of epithelial permeability [26-29] as well as in the regulation of pulmonary microvessels endothelium [26]. Moreover, TNF at higher concentrations leads to down-regulation of ZO-1 protein expression and disturbance in junction localization of ZO-1 protein and functional opening of tight junction barrier [29-31]. Base on this evidence, we have hypothesized that increased production of TNF-α might lead to structural and functional alterations in pulmonary TJ function in vivo as a result of acute lung inflammation induced by carrageenan in mice. Herein, we demonstrate that acute lung injury is associated with decreased expression and function of several TJ proteins in the lung epithelium. Moreover, we also demonstrate that Etanercept treatment attenuates TJ alteration associated with acute inflammation.

## Methods

### Animals

Mice (4–5 weeks old, 20–22 g) with a targeted disruption of the TNF-αR1 (TNF-α R1KO) and wild-type controls (TNF-αWT) were purchased from Jackson Laboratories (Charles River, Italy). The study was approved by the University of Messina Review Board for the care of animals. The animals were housed in a controlled environment and provided with standard rodent chow and water *ad libitum*. Animal care was in compliance with regulations in Italy (D.M. 116192), Europe (O.J. of E.C. L 358/1 12/18/1986) and USA (Animal Welfare Assurance No A5594-01, Department of Health and Human Services, USA).

### Experimental groups for Carrageenin-induced pleurisy

Mice were randomly allocated into the following groups: (i) *WT CAR group*. WT mice were subjected to carrageenan-induced pleurisy (*N *= 10); (ii) *CAR TNF-αR1KO group*. TNF-αR1KO mice were subjected to subjected to carrageenan-induced pleurisy (*N *= 10); (iii) *WT Sham group*. WT mice were subjected to the surgical procedures as the above groups except instead of carrageenan 100 μl of saline solution were administered to the mice (*N *= 10); (iv) *TNF-αR1KO Sham group*. TNF-αR1KO mice were subjected to the surgical procedures as the above groups except that instead of carrageenan 100 μl of saline solution were administered to the mice (*N *= 10); (v) *CAR WT+Etanercept group*. Same as CAR WT group except for the administration of Etanercept (5 mg/kg subcutaneously dissolved in saline solution) which was given at 2 h before the carrageenan injection (*N *= 10); (vi) *WT Sham +Etanercept group*. Same as the WT Sham group except for the administration of Etanercept (5 mg/kg subcutaneously dissolved in saline solution) which was given at 2 h before saline injection (*N *= 10);

### Carrageenan-induced pleurisy

Carrageenan-induced pleurisy was induced as previously described [32]. Mice were anesthetized with isoflurane and submitted to a skin incision at the level of the left sixth intercostals space. The underlying muscle was dissected and saline (0.2 ml) or saline containing 1% (w/v) λ-carrageenan (0.2 ml) was injected into the pleural cavity. The skin incision was closed with a suture and the animals were allowed to recover. At 4 h and 24 h after the injection of carrageenan, the animals were killed by inhalation of CO_2_. The chest was carefully opened and the pleural cavity rinsed with 2 ml of saline solution containing heparin (5 U/ml) and indomethacin (10 μg/ml). The exudate and washing solution were removed by aspiration and the total volume measured. Any exudate, which was contaminated with blood, was discarded. The amount of exudate was calculated by subtracting the volume injected (2 ml) from the total volume recovered. The leukocytes in the exudate were suspended in phosphate-buffer saline (PBS, 0.01 M, pH7.4) and counted with an optical microscope in a Burker's chamber after vital Trypan Blue staining.

### Histological examination

Lung biopsies were taken 4 h and 24 h after carrageenan injection. Tissues biopsies were fixed for 1 week in 10 % (w/v) PBS-buffered formaldehyde solution at room temperature, dehydrated using graded ethanol and embedded in Paraplast (Sherwood Medical, Mahwah, NJ, USA). Lung sections were then deparaffinized with xylene, stained with hematoxylin and eosin. All sections were studied using light microscopy (Dialux 22 Leitz).

### Measurement of cytokines

TNF-α production was evaluated in the pleural exudate and lung tissues at 4 h and 24 h after the induction of pleurisy by carrageenan injection as previously described [33]. The assay was carried out using a colorimetric commercial ELISA kit (Calbiochem-Novabiochem Corporation, Milan, Italy) with a lower detection limit of 10 pg/ml.

### Immunohistochemical localization of TNF-α, BAX- BCL-2 Claudin-2, Claudin-4, Claudin-5, ZO-I and β-catenin

At 4 and 24 h after carrageenan injection, tissues were fixed in 10% (w/v) PBS-buffered formaldehyde and 5 μm sections were prepared from paraffin embedded tissues. After deparaffinization, endogenous peroxidase was quenched with 0.3% (v/v) hydrogen peroxide in 60% (v/v) methanol for 30 min. Non-specific adsorption was minimized by incubating the section in 2% (v/v) normal goat serum in PBS for 20 min. Endogenous biotin or avidin binding sites were blocked by sequential incubation for 15 min with biotin and avidin (DBA, Milan, Italy), respectively. Sections were incubated overnight with 1) with anti-TNF-α antibody (Santa Cruz, 1:100 in PBS w/v,) or 3) with anti-Bax antibody (Santa Cruz, 1:50 in PBS v/v) or 4) with anti-Bcl-2 antibody (Santa Cruz 1:100 in PBS v/v). After deparaffinization, for *Claudin-2, Claudin-4, Claudin-5, ZO-I and β-catenin *detection, slices were treated with protease (type XIV, Sigma) (2 mg/ml) and for 10 min at 37°C. Detection of BCL-2 and Bax was carried out after boiling in citrate buffer, 0.01 M for 4 min. Sections were incubated overnight with polyclonal rabbit anti-claudin-2 *Claudin-4, Claudin-5, ZO-I and β-catenin *antibody (1:100 in PBS, w/v). Sections were washed with PBS and incubated with secondary antibody. Specific labeling was detected with a biotin-conjugated goat anti-rabbit IgG and avidin-biotin peroxidase complex (DBA, Milan, Italy). The counter stain was developed with DAB (brown color) and nuclear fast red (red background). To verify the binding specificity, some sections were also incubated with only the primary antibody (no secondary) or with only the secondary antibody (no primary). In these situations no positive staining was found in the sections indicating that the immunoreaction was positive in all the experiments carried out. Immunocytochemistry photographs (*N *= 5) were assessed by densitometry as previously described [34] by using Imaging Densitometer (AxioVision, Zeiss, Milan, Italy) and a computer program. In particular the densitometry analysis was carried out in section in which the lung was orientated in order to observe all the histological portions. In this type of section, is possible to evaluate the presence/absence or the alteration of the distribution pattern.

### Myeloperoxidase activity

Myeloperoxidase (MPO) activity, an indicator of polymorphonuclear leukocyte (PMN) accumulation, was determined as previously described [35]. At the specified time following injection of carrageenan, paw and lung tissues were obtained and weighed, each piece homogenized in a solution containing 0.5% (w/v) hexadecyltrimethyl-ammonium bromide dissolved in 10 mM potassium phosphate buffer (pH 7) and centrifuged for 30 min at 20,000 × g at 4°C. An aliquot of the supernatant was then allowed to react with a solution of tetramethylbenzidine (1.6 mM) and 0.1 mM hydrogen peroxide. The rate of change in absorbance was measured spectrophotometrically at 650 nm. MPO activity was defined as the quantity of enzyme degrading 1 μmol of peroxide/min at 37°C and was expressed in units per g of wet tissue.

### TUNEL Assay

TUNEL assay was conducted by using a TUNEL detection kit according to the manufacturer's instructions (Apotag, HRP kit DBA, Milan, Italy). Briefly, sections were incubated with 15 μg/ml proteinase K for 15 min at room temperature and then washed with PBS. Endogenous peroxidase was inactivated by 3% H_2_O_2 _for 5 min at room temperature and then washed with PBS. Sections were immersed in terminal deoxynucleotidyltransferase (TdT) buffer containing deoxynucleotidyl transferase and biotinylated dUTP in TdT buffer, incubated in a humid atmosphere at 37°C for 90 min, and then washed with PBS. The sections were incubated at room temperature for 30 min with anti-horseradish peroxidase-conjugated antibody, and the signals were visualized with diaminobenzidine.

From each biopsy, at least 4 bronchiolar profiles were evaluated under a light microscope at a ×20 magnification for TUNEL-positive cells. The percentage is calculated as the number of positive cells/total number of bronchial epithelial cells.

### Statistical evaluation

All values in the figures and text are expressed as mean ± standard error of the mean (s.e.m.) from 10 mice for each group. For the *in vivo *studies *n *represents the number of animals studied. In the experiments involving histology or immunohistochemistry, the figures shown are representative at least three experiments (histological or immunohistochemistry coloration) performed on different experimental days on the tissues section collected from all the animals in each group. The results were analyzed by one-way ANOVA followed by a Bonferroni's *post-hoc *test for multiple comparisons. A *p*-value of less than 0.05 was considered significant.

## Results

### Effects of TNF-α gene deletion and Etanercept administration on TNFα production

**Figure 1:**
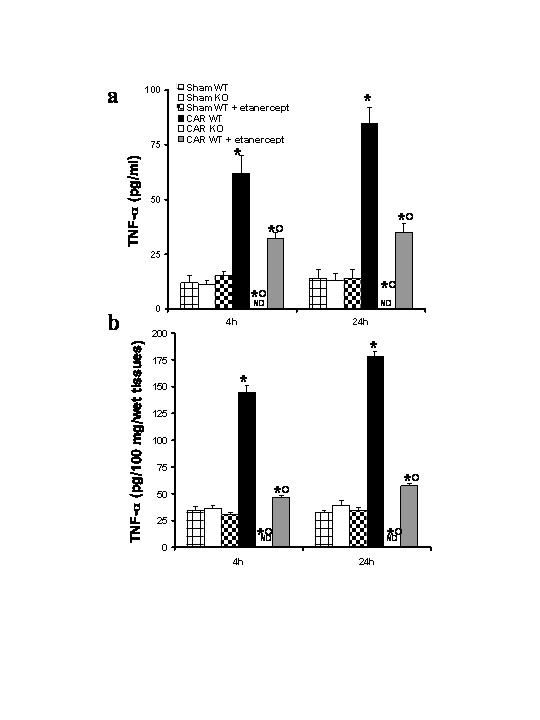
Effects of TNF-α gene deletion and Etanercept administration on TNFα levels. TNF-α production was evaluated in the pleural exudates (**a**) and lung tissues (**b**) collected at 4 h and 24 h after carrageenan administration using a colorimetric commercial ELISA kit. A significant production TNF-α was observed in pleural exudates (**a**) collected from WT mice. The absence of TNF-α receptor 1 gene in mice (TNF-αR1KO) as well as the treatment of WT mice with Etanercept significantly reduced the pleural exudate production of TNF-α. Similarly, a significant increase of the TNF-α (**b**) was observed in the lung tissues from carrageenan-injected WT mice at 4 and 24 hours after carrageenan. In the lung tissues from carrageenan-injected TNF-αR1KO mice as well as of WT mice which have received with Etanercept the TNF-α levels were significantly reduced in comparison to those of WT animals measured in the same conditions. Data are means ± SEM of 10 mice for each group. *P < 0.01 *vs*. SHAM; °*P *< 0.01 *vs*. carrageenan- WT group.

**Figure 2:**
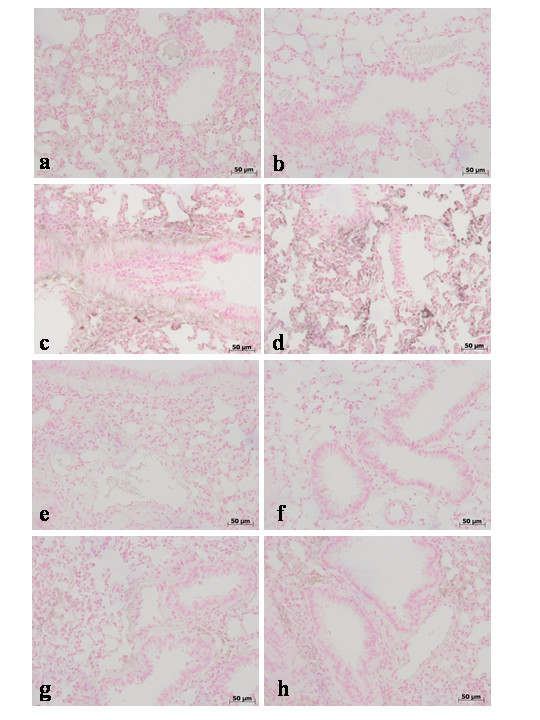
Immunohistochemical localization of TNF-α in the lung. No positive staining for TNF-α was observed in the lung tissues collected at 24 hours from sham WT mice (**a**) and from sham TNF-αR1KO mice (**b**). On the contrary, tissue sections obtained from WT animals at 4 h (**c**) and at 24 hours (**d**) after carrageenan injection demonstrate positive staining for TNF-α mainly localized in the infiltrated inflammatory cells, pneumocytes as well as in vascular wall. In carrageenan-injected TNF-αR1KO mice, no positive staining for TNF-α were observed in the lung tissues collected at 4 h (**e**) and at 24 hours (**f**). Similarly, the treatment of WT mice with Etanercept (5 mg/kg administered s.c. 2 h prior carrageenan) visibly and significantly reduced the positive staining for TNF-α in the infiltrated inflammatory cells, pneumocytes as well as in vascular wall in the lung tissues collected at 4 h (**g**) and at 24 hours (**h**). Figure is representative of at least 3 experiments performed on different experimental days.

**Figure 3:**
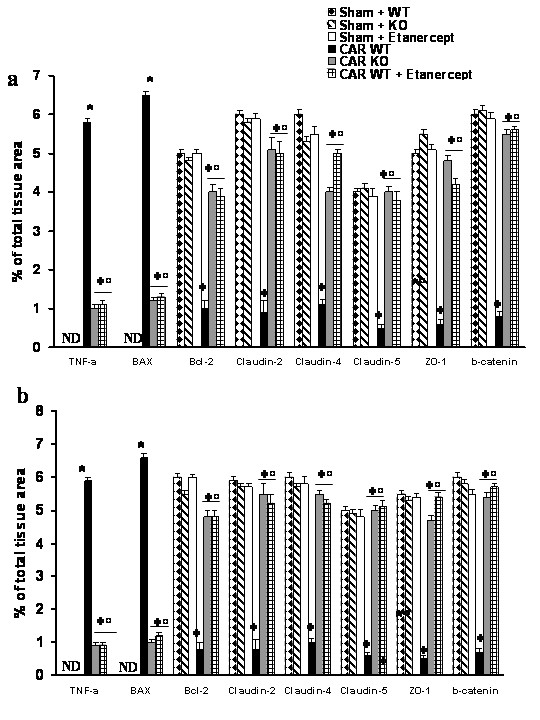
**Typical Densitometry evaluation**. Densitometry analysis of immunocytochemistry photographs (n = 5 photos from each sample collected from all mice in each experimental group) for TNF-α, Bax, Bcl-2 Claudin-2, Claudin-4, Claudin-5, ZO-I and β-catenin from lung tissues was assessed. The assay was carried out by using Imaging Densitometer (AxioVision, Zeiss, Milan, Italy) on a personal computer. Data are expressed as % of total tissue area. *P < 0.01 *vs*. SHAM; °*P*<0.01 *vs*. carrageenan- WT group ND: not detectable.

To test whether TNF-α gene may modulate the inflammatory process leading to structural and functional alterations in pulmonary TJ function in vivo, we analyzed the levels of this pro-inflammatory cytokine in TNF-αR1KO and WT mice. A substantial increase of TNF-α production was found in pleural exudates and in the lung tissues collected from WT mice at 4 h and 24 h after carrageenan injection (Fig 1). Pleural exudates and lung tissues production of TNF-α were significantly reduced in carrageenan-injected TNF-αR1KO mice as well as in WT mice treated with Etanercept (5 mg/kg administered s.c. 2 h prior carrageenan) (Fig. 1). Therefore, we also evaluate the TNF-α expression in the lung tissues by immunohistochemical detection. No positive staining for TNF-α was observed in the lung tissues collected at 4 h (**data not shown**) and at 24 hours from sham WT mice (Fig. 2a) and from sham TNF-αR1KO mice (Fig. 2b). On the contrary, tissue sections obtained from WT animals at 4 h (Fig. 2c**see densitometry analysis **Fig. 3a) and at 24 hours (Fig. 2d**see densitometry analysis **Fig. 3b) after carrageenan injection demonstrate positive staining for TNF-α mainly localized in the infiltrated inflammatory cells, pneumocytes as well as in vascular wall. In carrageenan-injected TNF-αR1KO mice, no positive staining for TNF-α were observed in the lung tissues collected at 4 h (Fig. 2e**see densitometry analysis **Fig. 3a) and at 24 hours (Fig. 2f**see densitometry analysis **Fig. 3b). Similarly, the treatment of WT mice with Etanercept (5 mg/kg administered s.c. 2 h prior carrageenan) visibly and significantly reduced the positive staining for TNF-α in the infiltrated inflammatory cells, pneumocytes as well as in vascular wall in the lung tissues collected at 4 h (Fig. 2g**see densitometry analysis **Fig. 3a) and at 24 hours (Fig. 2h**see densitometry analysis **Fig. 3a).

### Effects of TNF-α gene deletion and Etanercept administration on apoptosis in acute lung inflammation

**Figure 4:**
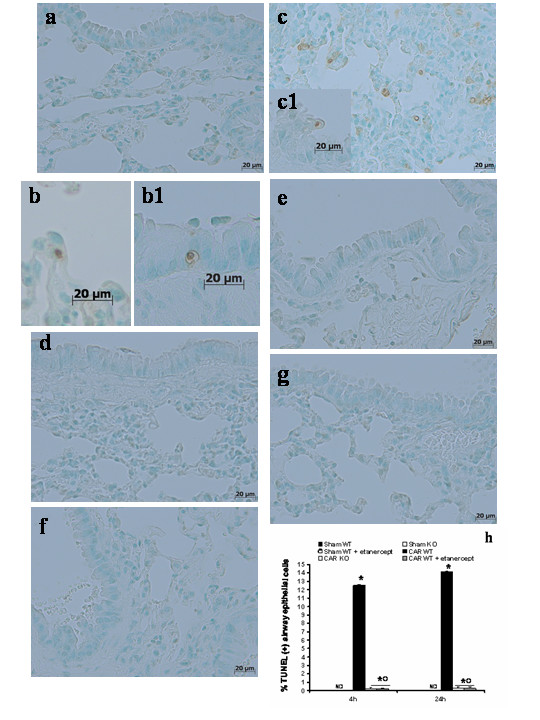
Effects of TNF-α gene deletion and Etanercept administration on apoptosis. Almost no apoptotic cells were detected in the lung from sham-treated (**a**) tissues collected at 24 hours. At 4 h (**bb1**) and 24 h (**cc1**) after the induction of lung inflammation by carrageenan administration, lung tissues demonstrated a marked appearance of dark brown apoptotic cells and intercellular apoptotic fragments. The presence of apoptotic cells or fragments were significantly reduced in the absence of a functional TNF-α R1 gene in TNF-αR1KO mice at 4 h (**d**) and at 24 hours (**e**) after carrageenan. Similarly, the treatment of WT mice with Etanercept (5 mg/kg administered sc 2 h prior carrageenan) visibly and significantly reduced the number of apoptotic cells or fragments in the lung tissues collected at 4 h (**f**) and at 24 hours (**g**). The graph (**h**) shows the mean ± SE of percentage TUNEL positive epithelial cells in biopsies from mice in all the experimental groups. *P < 0.01 *vs*. SHAM; °*P *< 0.01 *vs*. carrageenan- WT group ND: not detectable. Figure is representative of at least 3 experiments performed on different experimental days.

To test whether functional TNF-α gene plays a role on apoptosis in acute lung inflammation, we measured TUNEL-like staining in the lung tissues at 4 h and 24 h after carrageenan administration. Almost no apoptotic cells were detected in the lung from sham-treated (Fig. 4a**see (TUNEL)-positive cells count **Fig 4h). At 4 h (Fig. 4bb1**see (TUNEL)-positive cells count **Fig 4h) and 24 h (Fig. 4cc1**see (TUNEL)-positive cells count **Fig 4h) after the induction of lung inflammation by carrageenan administration, lung tissues demonstrated a marked appearance of dark brown apoptotic cells and intercellular apoptotic fragments. The presence of apoptotic cells or fragments were significantly reduced in the absence of a functional TNF-α R1 gene in TNF-αR1KO mice at 4 h (Fig. 4d**see (TUNEL)-positive cells count **Fig 4h) and at 24 hours (Fig. 4e**see (TUNEL)-positive cells count **Fig 4h) after carrageenan. Similarly, the treatment of WT mice with Etanercept (5 mg/kg administered sc 2 h prior carrageenan) visibly and significantly reduced the number of apoptotic cells or fragments in the lung tissues collected at 4 h (Fig. 4f**see (TUNEL)-positive cells count **Fig 4h) and at 24 hours (Fig. 4g**see (TUNEL)-positive cells count **Fig 4h).

Moreover, samples of lung tissue were taken at 4 and 24 h after carrageenan administration in order to determine the immunohistological staining for Bax and Bcl-2.

**Figure 5:**
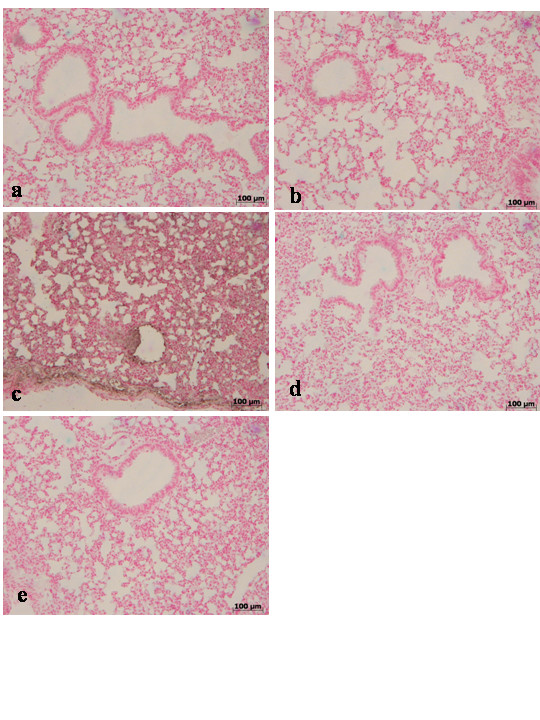
Immunohistochemical localization of Bax in the lung. Lung biopsies were taken 24 h after injection of carrageenan. No positive staining for Bax was observed in the lung tissues collected at 24 hours from sham WT mice (**a**) and from sham TNF-αR1KO mice (**b**). On the contrary, tissue sections obtained from WT animals at 24 hours (**c**) after carrageenan injection demonstrate positive staining for TNF-α mainly localized in the infiltrated inflammatory cells, pneumocytes as well as in vascular wall. In carrageenan-injected TNF-αR1KO mice, no positive staining for Bax were observed in the lung tissues collected at 24 hours (**d**). Similarly, the treatment of WT mice with Etanercept (5 mg/kg administered s.c. 2 h prior carrageenan) visibly and significantly reduced the positive staining for Bax in the infiltrated inflammatory cells, pneumocytes as well as in vascular wall in the lung tissues collected at 24 hours (**e**). Figure is representative of at least 3 experiments performed on different experimental days.

No positive staining for Bax was observed in the lung tissues collected at 4 h (**data not shown**) and at 24 hours from sham WT mice (Fig. 5a**see densitometry analysis **Fig. 3a) and from sham TNF-αR1KO mice (Fig. 5b**see densitometry analysis **Fig. 3b). On the contrary, tissue sections obtained from WT animals at 4 h (**data not shown see densitometry analysis **Fig. 3a) and at 24 hours (Fig. 5c**see densitometry analysis **Fig. 3b) after carrageenan injection demonstrate positive staining for Bax. In carrageenan-injected TNF-αR1KO mice, no positive staining for Bax were observed in the lung tissues collected at 4 h (**data not shown, see densitometry analysis **Fig. 3a) and at 24 hours (Fig. 5d**see densitometry analysis **Fig. 3b). Similarly, the treatment of WT mice with Etanercept (5 mg/kg administered sc 2 h prior carrageenan) reduced the degree of positive staining for Bax in the lung tissues at 4 h (**data not shown, see densitometry analysis **Fig. 3a) and at 24 hours (Fig. 5e**see densitometry analysis **Fig. 3b).

**Figure 6:**
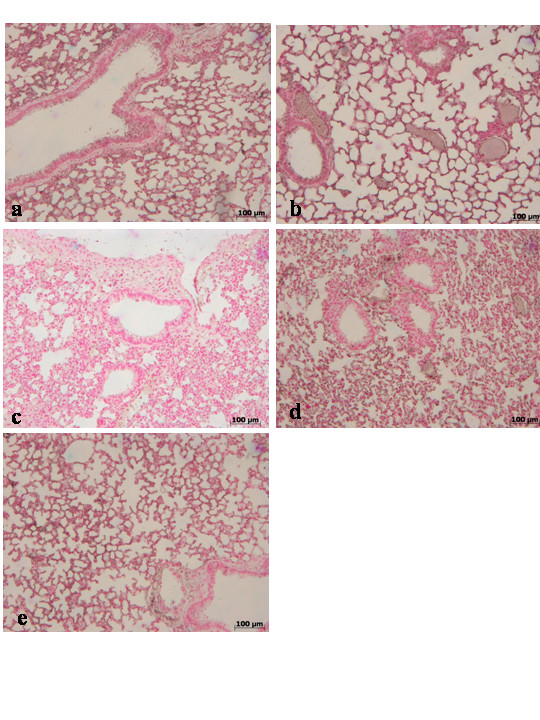
Immunohistochemical localization of Bcl-2 in the lung. Lung biopsies were taken 24 h after injection of carrageenan. Lung tissue sections collected at 24 hours from sham WT mice (**a**) and from sham TNF-αR1KO mice (**b**) demonstrated Bcl-2 positive staining while in the lung from carrageenan-treated WT mice the staining significantly reduced and at 24 hours (**c**). In carrageenan-injected TNF-αR1KO mice, a regular presence of Bcl-2 distribution was observed in the lung tissues collected at 24 hours (**d**). Similarly, the treatment of WT mice with Etanercept (5 mg/kg administered sc 2 h prior carrageenan) attenuated the loss of positive staining for Bcl-2 in the lung tissues at 24 hours (**e**). Figure is representative of at least 3 experiments performed on different experimental days.

In addition, lung tissue sections from sham WT mice (Fig. 6a**see densitometry analysis **Fig. 3b) and from sham TNF-αR1KO mice (Fig. 6b**see densitometry analysis **Fig. 3b) demonstrated Bcl-2 positive staining while in the lung from carrageenan-treated WT mice the staining significantly reduced at 4 h (**data not shown, see densitometry analysis **Fig. 3a) and at 24 hours (Fig. 6c**see densitometry analysis **Fig. 3a). In carrageenan-injected TNF-αR1KO mice, a regular presence of Bcl-2 distribution was observed in the lung tissues collected at 4 h (**data not shown, see densitometry analysis **Fig. 3a) and at 24 hours (Fig. 6d**see densitometry analysis **Fig. 3b). Similarly, the treatment of WT mice with Etanercept (5 mg/kg administered sc 2 h prior carrageenan) attenuated the loss of positive staining for Bcl-2 in the lung tissues at 4 h (**data not shown, see densitometry analysis **Fig. 3a) and at 24 hours (Fig. 6e, **see densitometry analysis **Fig. 3a).

### Lower levels of leukocyte extravasation and of tissue permeability correlate with a different modulation of TNF-α gene deletion and Etanercept administration on epithelian/endothelial function during pleurisy

**Figure 7:**
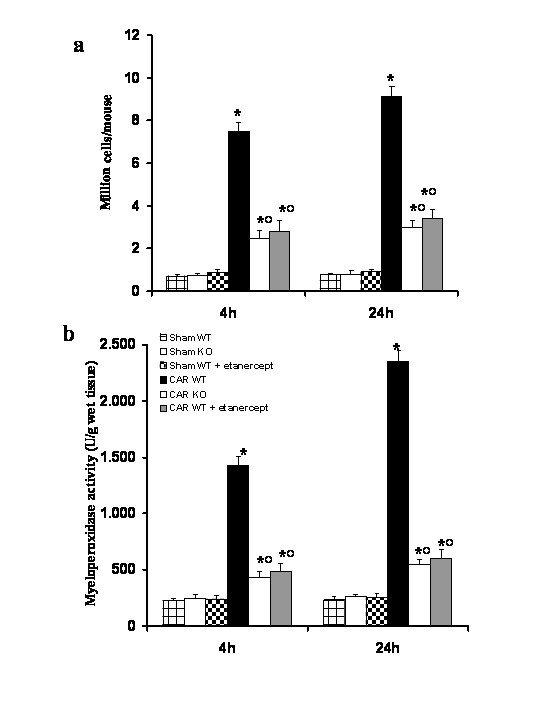
Effects of TNF-α gene deletion and Etanercept administration on carrageenan-induced accumulation of polymorphonuclear cells in pleural cavity (**a**) and neutrophils infiltration in the lung tissues (**b**). A significant polymorphonuclear cells infiltration (**a**) was observed in pleural cavity of WT mice at 4 h and 24 h after carrageenan administration. The absence of TNF-α receptor 1 gene in mice (TNF-αR1KO) as well as the treatment of WT mice with Etanercept significantly reduced the number of inflammatory cells in the pleural cavity (**a**) at 4 and 24 hours after carrageenan injection. In addition, at 4 and 24 hrs, the neutrophils infiltration by measurement of the activity of myeloperoxidase. Myeloperoxidase activity was significantly elevated at 4 h and 24 h after carrageenan administration in WT mice. In TNF-αR1KO mice lung myeloperoxidase activity was significantly reduced at 4 h and at 24 hours. Similarly, the treatment of WT mice with Etanercept (5 mg/kg administered s.c. 2 h prior carrageenan) significantly reduced the neutrophils infiltration in the lung tissues at 4 h and at 24 hours after carrageenan administration. Data are means ± SEM of 10 mice for each group. *P < 0.01 *vs*. SHAM; °*P *< 0.01 *vs*. carrageenan- WT group.

It is known that pro-inflammatory mediators modulate epithelial and endothelial function, which, in turn, favors the extravasation and recruitment of leukocytes further amplifying the inflammatory response. Therefore, we investigate the role of functional TNF-α gene on the polymorphonuclear cells (PMNs) in the pleural cavity. When compared to the number of cells collected from the pleural space from sham WT mice and sham TNF-αR1KO mice, injection of carrageenan induced a significant time dependent increase in the number of PMNs (Fig. 7a). The number of inflammatory cells (Fig. 7a) in the pleural cavity at 4 and 24 hours after carrageenan administration was significantly reduced in the absence of a functional TNF-αR1 gene in TNF-αR1KO mice. Similarly, treatment of WT mice with Etanercept (5 mg/kg administered s.c. 2 h prior carrageenan) significantly reduced the inflammatory cell infiltration in the pleaural cavity (Fig. 7a).

The important presence of inflammatory cells in the pleural cavity appeared to be correlated with the influx of leukocytes into the lung tissue. Therefore, we investigate the role of functional TNF-α gene on the neutrophils infiltration by measurement of the activity of myeloperoxidase. Myeloperoxidase activity was significantly elevated (p < 0.001) at 4 h and 24 h after carrageenan injection in WT mice (Fig. 7b). In lung from TNF-αR1KO mice myeloperoxidase activity was significantly reduced at 4 h and at 24 hours (p < 0.01) in comparison to those of WT animals (Fig. 7b). Similarly, the treatment of WT mice with Etanercept (5 mg/kg administered s.c. 2 h prior carrageenan) significantly reduced the neutrophils infiltration in the lung tissues at 4 h and at 24 hours after carrageenan-injection (Fig. 7b).

**Figure 8:**
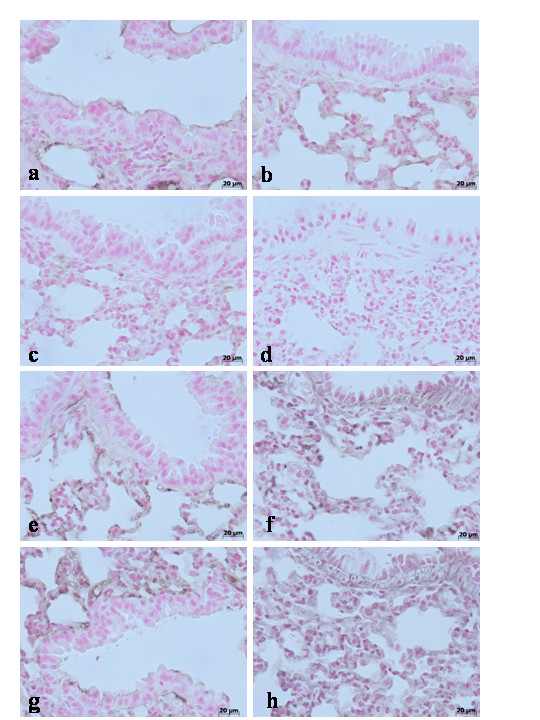
Immunohistochemical localization of ZO-1 in the lung. In lung tissue sections collected at 24 hours from sham WT mice (**a**) and from sham TNF-αR1KO mice (**b**), ZO-1 was uniformly and continuously distributed along the alveolar epithelium and the vascular endothelium. In contrast, a significant disruption of immuno-signal for ZO-1 was observed along the alveolar epithelium and the vascular endothelium in the lung sections of carrageenan-treated WT mice at 4 h (**c**) and at 24 hours (**d**) after carrageenan. In lungs of carrageenan-treated TNF-αR1KO mice, a more regular distribution pattern of ZO-1 along the alveolar epithelium and the vascular endothelium was found at 4 h (**e**) and at 24 hours (**f**), thus indicating that in carrageenan-treated TNF-αR1KO mice carrageenan treatment provokes a lower degree of disorganization of ZO-1. Similarly, the treatment of WT mice with Etanercept (5 mg/kg administered s.c. 2 h prior carrageenan) visibly and significantly reduced the disorganization of ZO-1 in the lung tissues collected at 4 h (**g**) and at 24 hours (**h**). Figure is representative of at least 3 experiments performed on different experimental days.

During the inflammatory process, tissue permeability is modified in part by changes in tight junctions [2,36]. Since ZO-1 is implicated in tight junction regulation and was used as a marker of cellular barrier integrity [37], we performed immunoistochemistry experiments to evaluate ZO-1 distribution in lung sections. Results indicate that in the lung tissues collected at 4 h (**data not shown see densitometry analysis **Fig. 3a) and at 24 hours from sham WT mice (Fig. 8a**see densitometry analysis **Fig. 3b) and from sham TNF-αR1KO mice (Fig. 8b**see densitometry analysis **Fig. 3b), ZO-1 was uniformly and continuously distributed along the alveolar epithelium and the vascular endothelium. In contrast, a significant disruption of immuno-signal for ZO-1 was observed along the alveolar epithelium and the vascular endothelium in the lung sections of carrageenan-treated WT mice at 4 h (Fig. 8c**see densitometry analysis **Fig. 3a) and at 24 hours (Fig. 8d**see densitometry analysis **Fig. 3b) after carrageenan. In lungs of carrageenan-treated TNF-αR1KO mice, a more regular distribution pattern of ZO-1 along the alveolar epithelium and the vascular endothelium was found at 4 h (Fig. 8e**see densitometry analysis **Fig. 3a) and at 24 hours (Fig. 8f**see densitometry analysis **Fig. 3b), thus indicating that in carrageenan-treated TNF-αR1KO mice carrageenan treatment provokes a lower degree of disorganization of ZO-1. Similarly, the treatment of WT mice with Etanercept (5 mg/kg administered s.c. 2 h prior carrageenan) visibly and significantly reduced the disorganization of ZO-1 in the lung tissues collected at 4 h (Fig. 8g**see densitometry analysis **Fig. 3a) and at 24 hours (Fig. 8h**see densitometry analysis **Fig. 3b).

**Figure 9:**
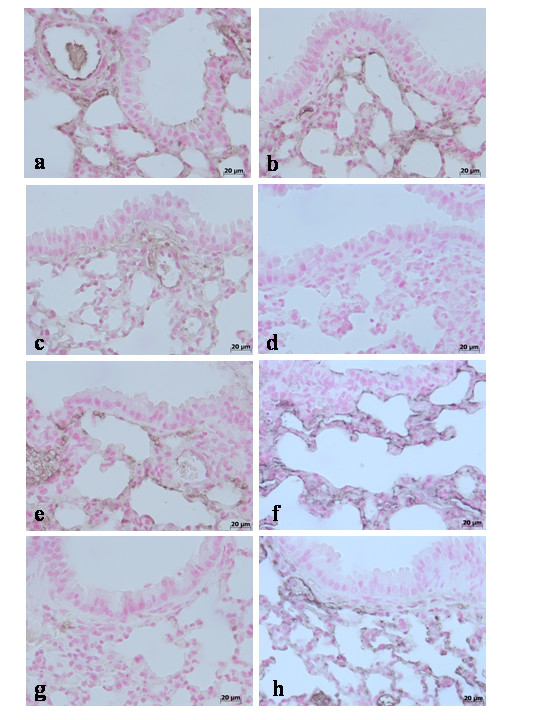
Immunohistochemical localization of Claudin-2 in the lung. In lung tissue sections collected at 24 hours from sham WT mice (**a**) and from sham TNF-αR1KO mice (**b**), Claudin-2 were uniformly and continuously distributed along the alveolar epithelium and the vascular endothelium. On the contrary, a significant disruption of immunohistochemical localization signal for Claudin-2 was observed in the lung tissues sections of carrageenan-treated WT mice at 4 h (**c**) and at 24 hours (**d**) after carrageenan. In lungs of carrageenan-treated TNF-αR1KO mice was found less irregular distribution pattern of Claudin-2 at 4 h (**e**) and at 24 hours (**f**) after carrageenan. Similarly, the treatment of WT mice with Etanercept (5 mg/kg administered s.c. 2 h prior carrageenan) were able to prevent carrageenan-induced distribution alteration of Claudin-2 at 4 h (**g**) and at 24 hours (**h**) after carrageenan. Figure is representative of at least 3 experiments performed on different experimental days.

**Figure 10:**
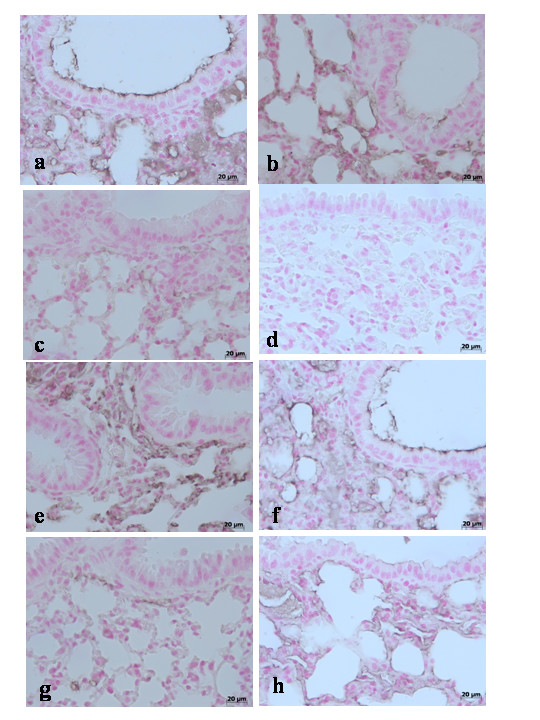
Immunohistochemical localization of Claudin-4 in the lung. In lung tissue sections collected at 24 hours from sham WT mice (**a**) and from sham TNF-αR1KO mice (**b**), Claudin-4 were uniformly and continuously distributed along the alveolar epithelium and the vascular endothelium. On the contrary, a significant disruption of immunohistochemical localization signal for Claudin-4 was observed in the lung tissues sections of carrageenan-treated WT mice at 4 h (**c**) and at 24 hours (**d**) after carrageenan. In lungs of carrageenan-treated TNF-αR1KO mice was found less irregular distribution pattern of Claudin-4 at 4 h (**e**) and at 24 hours (**f**) after carrageenan. Similarly, the treatment of WT mice with Etanercept (5 mg/kg administered s.c. 2 h prior carrageenan) were able to prevent carrageenan-induced distribution alteration of Claudin-4 at 4 h (**g**) and at 24 hours (**h**) after carrageenan. Figure is representative of at least 3 experiments performed on different experimental days.

**Figure 11:**
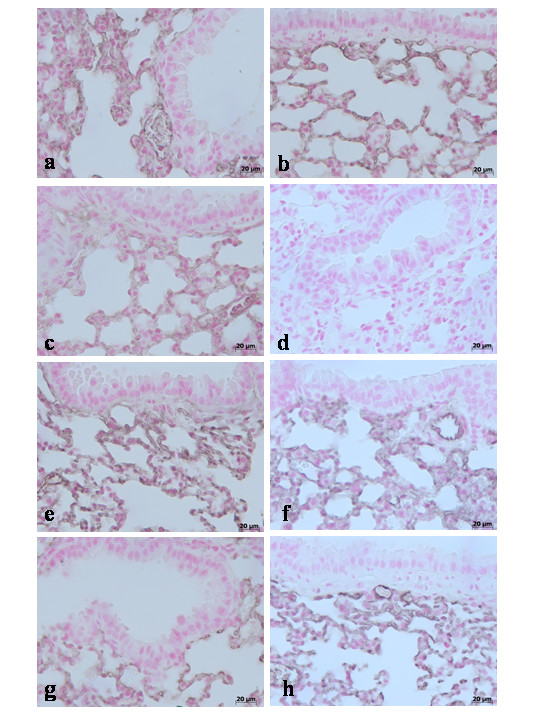
Immunohistochemical localization of Claudin-5 in the lung. In lung tissue sections collected at 24 hours from sham WT mice (**a**) and from sham TNF-αR1KO mice (**b**), Claudin-5 were uniformly and continuously distributed along the alveolar epithelium and the vascular endothelium. On the contrary, a significant disruption of immunohistochemical localization signal for Claudin-5 was observed in the lung tissues sections of carrageenan-treated WT mice at 4 h (**c**) and at 24 hours (**d**) after carrageenan. In lungs of carrageenan-treated TNF-αR1KO mice was found less irregular distribution pattern of Claudin-5 at 4 h (**e**) and at 24 hours (**f**) after carrageenan. Similarly, the treatment of WT mice with Etanercept (5 mg/kg administered s.c. 2 h prior carrageenan) were able to prevent carrageenan-induced distribution alteration of Claudin-5 at 4 h (**g**) and at 24 hours (**h**) after carrageenan. Figure is representative of at least 3 experiments performed on different experimental days.

**Figure 12:**
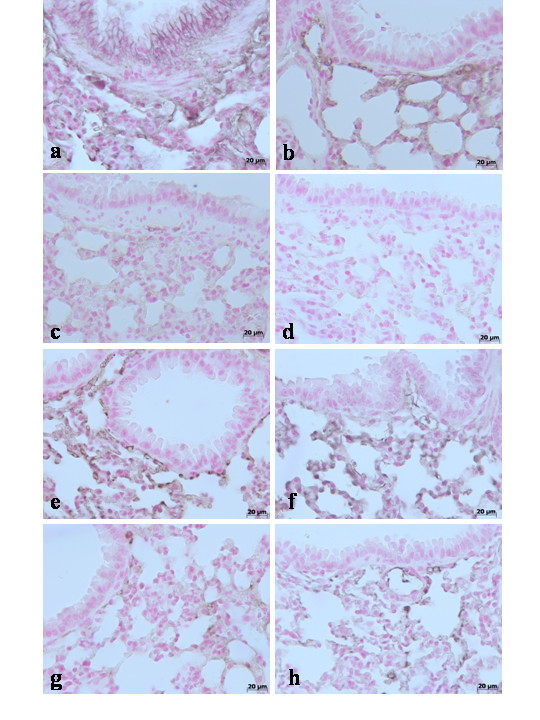
Immunohistochemical localization of β-catenin in the lung. In lung tissue sections collected at 24 hours from sham WT mice (**a**) and from sham TNF-αR1KO mice (**b**), β-catenin were uniformly and continuously distributed along the alveolar epithelium and the vascular endothelium. On the contrary, a significant disruption of immunohistochemical localization signal for β-catenin was observed in the lung tissues sections of carrageenan-treated WT mice at 4 h (**c**) and at 24 hours (**d**) after carrageenan. In lungs of carrageenan-treated TNF-αR1KO mice was found less irregular distribution pattern of β-catenin at 4 h (**e**) and at 24 hours (**f**) after carrageenan. Similarly, the treatment of WT mice with Etanercept (5 mg/kg administered s.c. 2 h prior carrageenan) were able to prevent carrageenan-induced distribution alteration of β-catenin at 4 h (**g**) and at 24 hours (**h**) after carrageenan. Figure is representative of at least 3 experiments performed on different experimental days.

In addition, results indicate that in the lung tissues collected at 4 h (**data not shown see densitometry analysis **Fig. 3a) and at 24 hours from sham WT mice and from sham TNF-αR1KO mice, Claudin-2 (Fig. 9a,b**respectively see densitometry analysis **Fig. 3b), Claudin-4 (Fig. 10a,b**respectively see densitometry analysis **Fig. 3b), Claudin-5 (Fig. 11a,b**respectively see densitometry analysis **Fig. 3b) and β-catenin (Fig. 12a,b**respectively, see densitometry analysis **Fig. 3b) were uniformly and continuously distributed along the alveolar epithelium and the vascular endothelium. On the contrary, a significant disruption of immunohistochemical localization signal for Claudin-2 (Fig. 9cd**respectively**), Claudin-4 (Fig. 10bc**respectively**), Claudin-5 (Fig. 11cd**respectively**) and β-catenin (Fig. 12cd**respectively) **was observed in the lung tissues sections of carrageenan-treated WT mice at 4 h (**see densitometry analysis **Fig. 3a) and 24 h (**see densitometry analysis **Fig. 3b) after carrageenan. In lungs of carrageenan-treated TNF-αR1KO mice was found less irregular distribution pattern of Claudin-2 (Fig. 9ef**respectively)**, Claudin-4 (Fig. 10de**respectively)**, Claudin-5 (Fig. 11ef**respectively) **and β-catenin (Fig. 12ef**respectively**) at 4 h (**see densitometry analysis **Fig. 3a) and at 24 hours (**see densitometry analysis **Fig. 3a). Similarly, the treatment of WT mice with Etanercept (5 mg/kg administered s.c. 2 h prior carrageenan) were able to prevent carrageenan-induced distribution alteration of Claudin-2 (Fig. 9gh**respectively**), Claudin-4 (Fig. 10ef**respectively**), Claudin-5 (Fig. 11gh**respectively**) and β-catenin (Fig. 12gh**respectively**) at 4 h (**see densitometry analysis **Fig. 3a) and at 24 hours (**see densitometry analysis **Fig. 3a).

A massive increase in the lung permeability was observed at 4 and 24 h after carrageenan administration, as evidenced by a marked increase in the pleural exudates formation in WT mice (Fig. 12). Significant reduction of pleural exudates formation was observed at 4 and 24 h after carrageenan administration in carrageenan-treated TNF-αR1KO mice as well as increase in the carrageenan-treated WT mice which have received Etanercept (5 mg/kg administered s.c. 2 h prior carrageenan) (Fig. 12).

## Discussion

The gas exchanging air-blood barriers in the lung are composed of the microvascular pulmonary endothelium and the epithelial lining of the alveoli, which form three separate compartments: blood, interstitium, and the alveolar space. Acute lung injury and its most severe form, the acute respiratory distress syndrome (ARDS), are conditions characterized by a diffuse, intense inflammatory process, by damage to both endothelial and epithelial cell barriers, resulting in marked extravasation of vascular fluid [38]. The filling of alveolar spaces by proteinaceous edema fluid and inflammatory cells leads to severe hypoxemia and respiratory failure [38]. The normal functioning of the lung depends on the establishment and maintenance of a milieu in the alveolar space that is distinct from the composition of the subepithelial compartment. This process depends on the formation and proper functioning of specialized structures, called tight junctions (TJ), between adjacent cells making up the epithelial sheet. The TJ is a complex of several integral membrane proteins and peripheral membrane proteins that interact strongly with the cytoskeleton [39]. Integral membrane proteins involved in TJ formation include occludin and members of a large class of proteins called claudins [39]. These proteins contain four transmembrane domains and are thought to be the points of cell-to-cell contact within the TJ [40]. Cosedimentation assays of TJ proteins suggest that there is a strong interaction between occludin and another protein associated with TJ formation, zonula occludens (ZO)-1 [41,42]. ZO-1 has been shown to interact with the cytoplasmic tails of occludin and the claudins. In addition, ZO-1 interacts with two additional members of the membrane-associated guanylate kinase family of proteins, ZO-2 and ZO-3 [43]. The TJ serves as a fence that differentiates the plasma membrane into apical and basolateral domains [44]. TJs also serve as a regulated semipermeable barrier that limits the passive diffusion of solutes across the paracellular pathway between adjacent cells [45]. These properties of TJs, in combination with transcellular vectorial transport processes, generate distinct internal environments in the opposing compartments.

Recently, we have demonstrated, using an experimental model of colitis, that the structure and function of TJs in the intestinal epithelial is dependent of the production of proinflammatory cytokines (e.g. TNF-α) [34]. In particular in one study, we have showed that the structure and function of TJs in the ileum were altered in mice at 4 days after the induction of experimental colitis [34]. These changes were ameliorated when mice were treated with Thalidomide. In the study reported here, we obtained similar results and showed that the structure and function of TJs in the lung were altered in mice injected 4 and 24 h earlier with carrageenan. Moreover, these changes were ameliorated by treatment with Etanercept, a TNF-α soluble receptor construct, as well as in mice with a targeted deletion of the TNF-αR1 gene. As a result of these efforts, we point out that acute lung inflammation induced alterations in the function of lungs, is due, at least in part, to massive changes in the expression and localization of key TJ proteins. Furthermore, we propose that excessive TNF-α production is an important component of this process.

What is then the mechanism by which inhibition of TNF-α induced the structure and function of TJs alteration in acute inflammation caused by carrageenan?

There is good evidence that TNF-α help to propagate the extension of a local or systemic inflammatory process [46,47]. We confirm here that carrageenan injection a substantial increase of TNF-α in the exudates and lung tissues which likely contribute in different capacities to the evolution of acute inflammation. As expected, the TNF-α levels were abolished in TNF-αR1KO mice and significantly reduced in the TNF-α R1WT mice treated with Etanercept.

Moreover various study have clearly point out that the immune system plays an important role in modulating epithelial permeability in different organs such us gut and lung [48,49]. The regulation of epithelial barrier function by cytokines has been studied most extensively in the gut using different epithelial cell line. For examples some studies have demonstrated that two cytokines, interferon-γ (IFNγ) and tumor necrosis factor-α (TNFα), decreased barrier function of cultured intestinal epithelial monolayers [50,51]. Moreover, incubation of intestinal epithelial cell monolayers with both IFNγ and TNFα leads to reorganization of many tight junction proteins, including ZO-1, junction adhesion molecule 1, occludin, claudin-1, and claudin-4 [27]. The effects of pro-inflammatory cytokines on lung epithelial barrier function were studied using lung epithelial cells. In a recent study Ahdieh and colleagues have clearly demonstrated Th1 and Th2 cytokines have distinct activities in lung epithelial cells that are consistent with the role of these cytokines in lung inflammation (e.g. asthma) In particular, IL-4 and IL-13 disrupt epithelial barrier function and wound healing, both of which would be expected to exacerbate inflammation. In contrast, IFN-γ enhances epithelial barrier function and wound healing, which would contribute to a reduction in inflammation [49].

In the present study, we also demonstrate that carrageenan administration to mice induced an alteration of immunohistochemical localization signal for ZO-1, Claudin-2, Claudin-4, Claudin-5 and β-catenin at 4 h and 24 h after carrageenan. The genetic or pharmacological inhibition of TNF-α abolished the alteration of tight junction permeability. Thus, a key step in the pathogenesis of acute lung injury may be myosin light chain kinase activation by TNFα, leading to epithelial barrier dysfunction. There is extensive literature examining the consequences of specific proinflammatory cytokines, particularly tumor necrosis factor (TNF)-α, on the induction of apoptosis. An important biologic effect of TNF-α is the induction of apoptosis through its interaction with its receptor, TNFR. However, the same TNF-TNFR ligand-receptor interaction can also activate NF-κB [52]. Activation of the Fas ligand is another major signaling pathway that leads to apoptosis. In bronchial epithelial cells, ligation of Fas stimulates both apoptosis and expression of IL-8 [53]. Thus, the activation of proinflammatory signaling pathways can elicit apoptotic responses as well as the expression of cytokines. The response of the normal airway epithelial cell to inflammatory stimuli may consist of activation of both apoptotic and proinflammatory cascades [52]. However, in diseases such as acute lung injury, in which there appears to be an excessive inflammatory response, the balance between the activation and suppression of these potentially opposing pathways may be abnormal. In the present study, using the tunnel coloration we have clearly confirmed that TNF-α plays an important role in the induction of apoptosis during acute lung injury and that the treatment with etanercept and genetic inhibition of TNF-αR1 attenuates the degree of apoptosis. Moreover, it is well known that Bax, a pro-apoptotic gene, plays an important role in developmental cell death (34). Similarly, it has been shown that the overexpression of Bcl-2, a known antiapoptotic factor, significantly reduced the bronchial as well as intestinal epithelial apoptosis [54,55]. Base on these evidences, we have identified proapoptotic transcriptional changes, including upregulation of proapoptotic Bax and down regulation of antiapoptotic Bcl-2 by immunohystochemical staining. We report in the present study for the first time that the treatment with etanercept and genetic inhibition of TNF-α R1prevents the lost of the antiapoptotic way and reduced the proapoptotic pathway activation with a mechanism still to discover.

Further studies are needed to address this point. Thus, we propose the following cycle: Inflammation -> TNF-α-> endothelial/epithelial TJ alteration -> PMN infiltration -> more pro-inflammatory mediator release -> organ damage. The confirmation of this proposed feedback cycle, however, requires further investigation. In conclusion, we have demonstrated *in vivo *that the pharmacological inhibition of TNF-α by Etanercept attenuates the development of TJ alteration in acute inflammation in mice.
